# From Simulation to Reality: Predicting Torque With Fatigue Onset via Transfer Learning

**DOI:** 10.1109/TNSRE.2024.3465016

**Published:** 2024-10-07

**Authors:** Kalyn M. Kearney, Tamara Ordonez Diaz, Joel B. Harley, Jennifer A. Nichols

**Affiliations:** J. Crayton Pruitt Family Department of Biomedical Engineering, University of Florida, Gainesville, FL 32611; J. Crayton Pruitt Family Department of Biomedical Engineering, University of Florida, Gainesville, FL 32611; Department of Electrical and Computer Engineering, University of Florida, Gainesville, FL 32603 USA.; J. Crayton Pruitt Family Department of Biomedical Engineering, University of Florida, Gainesville, FL 32611

**Keywords:** Artificial neural networks, electromyography, muscle fatigue, simulations, transfer learning, upper extremity

## Abstract

Muscle fatigue impacts upper extremity function but is often overlooked in biomechanical models. The present work leveraged a transfer learning approach to improve torque predictions during fatiguing upper extremity movements. We developed two artificial neural networks to model sustained elbow flexion: one trained solely on recorded data (i.e., direct learning) and one pre-trained on simulated data and fine-tuned on recorded data (i.e., transfer learning). We simulated muscle activations and joint torques using a musculoskeletal model and a muscle fatigue model (n = 1,701 simulations). We also recorded static subject-specific features (e.g., anthropometric measurements) and dynamic muscle activations and torques during sustained elbow flexion in healthy young adults (n = 25 subjects). Using the simulated dataset, we pre-trained a long short-term memory neural network (LSTM) to regress fatiguing elbow flexion torque from muscle activations. We concatenated this pre-trained LSTM with a feedforward architecture, and fine-tuned the model on recorded muscle activations and static features to predict elbow flexion torques. We trained a similar architecture solely on the recorded data and compared each neural network’s predictions on 5 leave-out subjects’ data. The transfer learning model outperformed the direct learning model, as indicated by a decrease of 24.9% in their root-mean-square-errors (6.22 Nm and 8.28 Nm, respectively). The transfer learning model and direct learning model outperformed analogous musculoskeletal simulations, which consistently underpredicted elbow flexion torque. Our results suggest that transfer learning from simulated to recorded datasets can decrease reliance on assumptions inherent to biomechanical models and yield predictions robust to real-world conditions.

## I. INTRODUCTION

MUSCLE fatigue, the exercise-induced reduction in a muscle’s force-generating capacity [1], can significantly influence biomechanical outcomes. When unaccounted for, muscle fatigue can limit the effectiveness of medical devices and impede athletic performance. For example, the shifts in muscle activations that result from muscle fatigue induce errors in myoelectric controllers [2], thereby hindering the utility of exoskeletons [3] and prostheses [4]. Furthermore, muscle fatigue causes shifts in postural strategies [5] and increases the likelihood of sports-related injuries [6], [7].

The wide-ranging implications of muscle fatigue have prompted researchers to model its effects on biomechanical systems. A multifaceted phenomenon, muscle fatigue has been modeled on multiple scales, from motor units [8] to the joint level [9], using techniques rooted in physics (e.g., [10]). Many factors can decrease a muscle’s force capacity during exercise [11], [12], [13], challenging the parameterization of complex physics-based models. For example, the model developed by Callahan et al. [10] employed a holistic approach, using the neural, biochemical, morphological, and physiological properties of skeletal muscle to predict neural activations, forces, and intracellular metabolic perturbations during fatiguing contractions. Such models provide important insights into the mechanisms that drive fatigue. However, the assumptions needed to parameterize and design complex physics-based models limit predictive performance.

Circumventing physical and subject-specific assumptions, machine learning models have also been employed to capture the effects of muscle fatigue [14]. Machine learning models have been developed to predict fatigue onset using data collected from wearable devices, such as motion (e.g., [15]) or electromyographic (EMG) data (e.g., [16]). Like physics-based models, machine learning models of muscle fatigue range in complexity. For example, Zhang et al. [17] used support vector machines, a traditional machine learning model, to classify muscle fatigue using lower extremity kinematic and kinetic data. Given the complexity of real-world biomechanical systems, more recent works have shifted to predicting the effects of muscle fatigue via deep learning (e.g., [18]). Unlike many traditional machine learning models, deep models can capture complex non-linear relationships in high dimensional datasets, thereby providing enhanced generalizability to unseen data.

Despite the prevalence of muscle fatigue models, limitations to predictive performance continue to stymie the progress of assistive and rehabilitative technologies. A notable challenge is that muscle fatigue introduces complex non-linear behaviors into biomechanical systems that are often difficult to model accurately. In part, this complexity is difficult to model due to the time and monetary expenses associated with collecting high-fidelity biomechanical data. These challenges are exacerbated in the upper extremity, where EMG crosstalk is difficult to avoid [19] and the high number of degrees of freedom increase model complexity. To leverage the strengths of both physics-based and machine learning models, some works have employed a strategy known as transfer learning, or the process of training a machine learning model on one task and repurposing this knowledge for another task (e.g., [20], [21], [22], [23]). As exemplified by our previous work [24], pre-training artificial neural networks (ANNs) with large, simulated datasets can improve the predictive performance of biomechanical machine learning models. A similar transfer learning approach may enhance predictions involving the effects of muscle fatigue.

Our objective was to improve the prediction of torque in fatiguing upper extremity movements. We chose to predict torque as it is correlated to the decline in muscle force caused by fatigue [25]. Furthermore, predicting changes in torque is valuable for the design of interventions to mitigate the impact of muscle fatigue; for example, myoelectric controllers equipped with a fatigue model could compensate for the effects of fatigue, enhancing device performance (e.g., [26]). Towards our objective, we developed a model of elbow flexion via transfer learning. Specifically, we pre-trained an ANN using simulated data and fine-tuned the ANN using recorded data. We also developed a direct learning model by training an ANN solely on recorded data. By comparing the predictions of each ANN on a leave out set, we elucidated the utility of simulated datasets for informing models robust to the onset of muscle fatigue. Lastly, we compared each ANN’s predictions to that of analogous musculoskeletal simulations, elucidating the benefits of our machine learning approaches relative to a standard physics-based approach.

## II. METHODS

**Fig. 1.**
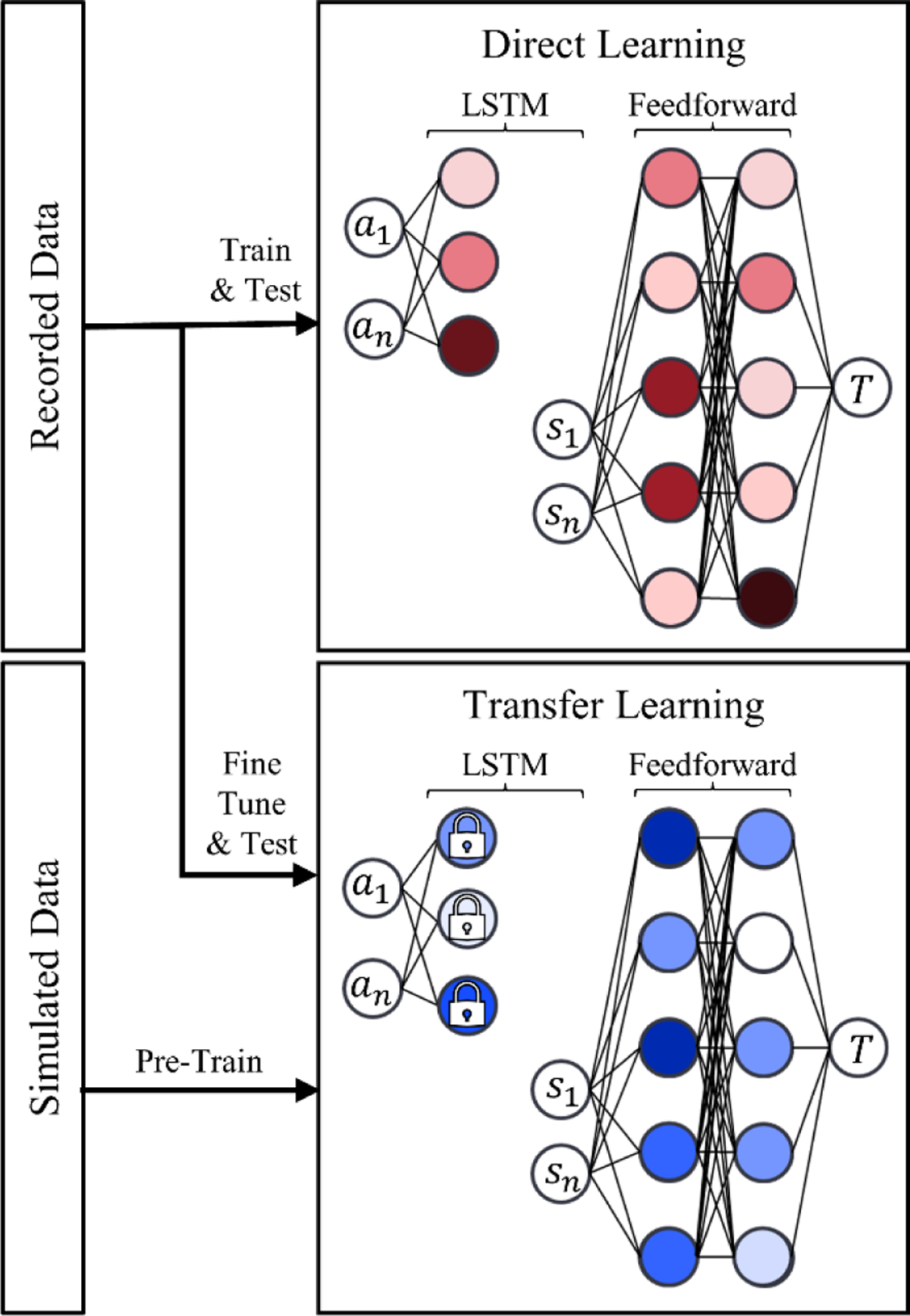
Overview of transfer learning approach and its validation. Simulated data were used to pre-train an ANN comprised of LSTM and feedforward layers. Muscle activations (denoted *a*_1_ through *a*_*n*_) were inputs to LSTM layers, and static subject-specific features (denoted *s*_1_ through *s*_*n*_) were inputs to the feedforward layers. The output of each ANN was elbow flexion torque, *T*. The pre-trained ANN was then fine tuned using recorded data, resulting in our transfer learning model (bottom). We trained a similar model solely of recorded data, resulting in our direct learning model (top). Each model was then evaluated using a leave out set, and the quality of their prediction were compared.

To evaluate the utility of transfer learning for modeling sustained elbow flexion, we developed and compared results from both a transfer learning model and direct learning model using PyTorch v. 2.0.1 [27]. Simulated and recorded datasets of sustained elbow flexion were used to train and validate these ANNs. Briefly, we simulated elbow flexion for healthy young adults using a generic elbow model [28] and a muscle fatigue model [29] in OpenSim v. 4.1 [30], [31]. As part of an IRB-approved study (University of Florida IRB #202202263), we also recorded sustained elbow flexion at 80% maximum voluntary contraction (MVC) in 25 healthy young adults. Both ANNs were then developed and evaluated on a leave-out set (Fig. 1). Each ANN used inputs of dynamic muscle activations and static subject-specific features (e.g., anthropometric measurements) to predict elbow flexion torques over time. Lastly, we ran a forward dynamic simulation for each subject in the leave-out set, and compared the errors of the transfer learning model, direct learning model, and forward dynamic simulations. The data required for reproducing our results are freely available at https://simtk.org/projects/transferlearnue.

### A. Simulated Dataset

**Fig. 2.**
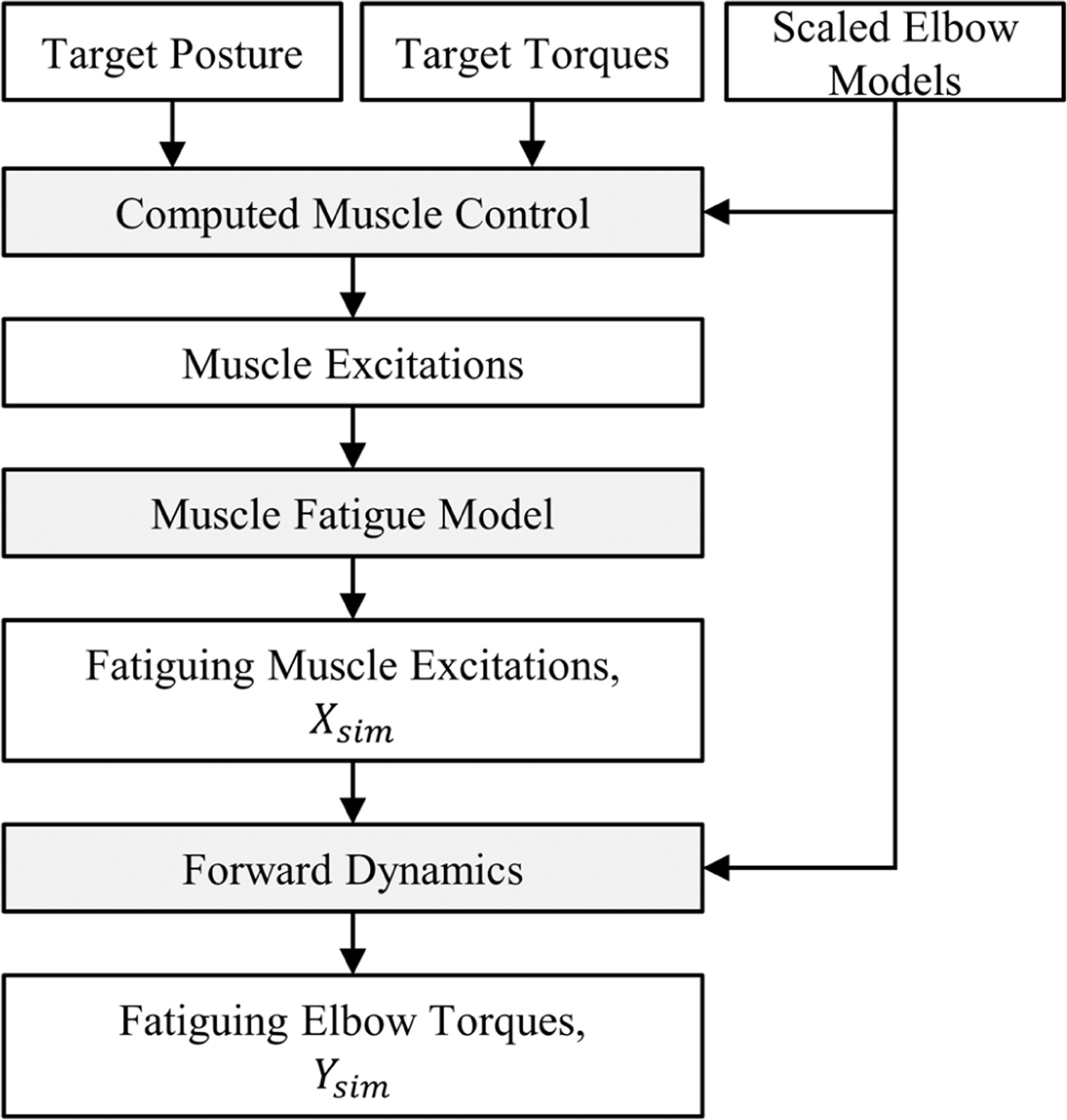
Overview of simulation pipeline. Scaled elbow models were fed into computed muscle control simulations defined with a static target posture and varying target forces. The resulting muscle excitations were then processed through a muscle fatigue model, before being used in forward dynamic simulations. The resulting fatiguing muscle excitations, *X*_*sim*_, and elbow torques, *Y*_*sim*_, were the inputs and outputs, respectively, used to pre-train transfer learning model.

**Fig. 3.**
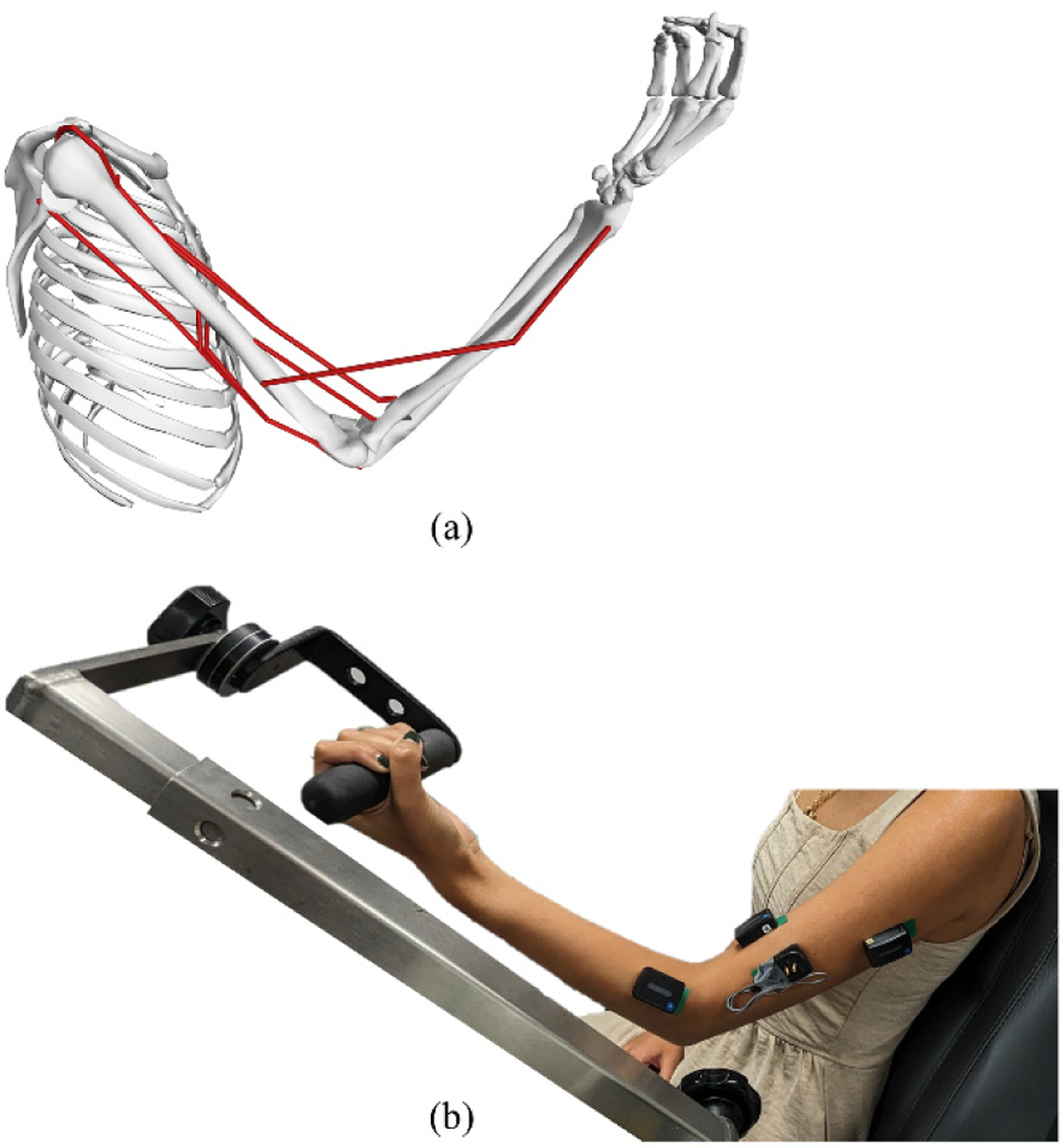
Generic musculoskeletal model [28] used to simulate elbow flexion (a) and experimental setup used to record elbow flexion (b).

To pre-train the transfer learning model, we simulated a dataset that represented elbow flexion with the onset of muscle fatigue (Fig. 2). We scaled a generic elbow model [28] using randomly sampled masses and heights [32] to generate 300 musculoskeletal models that represented young adults ranging from 5^th^ to 95^th^ percentile in size and 20–29 years of age. The musculoskeletal model included 7 muscle-tendon actuators: medial, lateral, and long heads of the *triceps* (TRI), long and short heads of the *biceps* (BIC), *brachialis* (BRA), and *brachioradialis* (BRD). Excitations for these actuators were generated via computed muscle control (CMC) simulations that varied in torque, targeting a 1.5 second linear ramp, which started at 10 Nm and peaked between 30 Nm and 90 Nm, adjusted in increments of 10 Nm, before plateauing for an additional 1.5 seconds. Each CMC simulation represented isometric elbow flexion, maintaining a target posture of 45° shoulder flexion, 90° elbow flexion, and forearm supination (Fig. 3a). Retrieving only simulations that converged (1,701 of 2,100 simulations), we extrapolated the final state (holding torque constant) to extend each simulation to 30 seconds, or the approximate time to exhaustion for an 80% MVC in healthy young adults [33]. The resulting simulated dataset contained sets of pre-fatigue muscle excitations representing 243 healthy young adults.

To simulate the effects of muscle fatigue, we processed the pre-fatigue muscle excitations from CMC using a muscle fatigue model [29]. This 3-compartment model represents a muscle’s motor units in active, rested, and fatigued states. Additionally, this fatigue model does not require any subject-specific parameters and has already been parameterized for the elbow [34]. Based on this prior work, we adopted fatigue rate and recovery rates of 0.00912 and 0.00094 (both unitless), respectively. In a pipeline similar to that employed by Samaan et al. [35], the pre-fatigue muscle excitations were processed through the fatigue model (Fig. 2). The resulting fatigued excitations were then used as inputs to forward dynamic simulations, from which elbow flexion torques were calculated. Lastly, each simulation was interpolated to contain 300 time instances (i.e., a frequency of 10 Hz). The resulting simulated dataset contained 1,701 sets of muscle excitations and elbow torques which capture the effects of fatigue for 30 seconds of sustained, isometric elbow flexion.

### B. Recorded Dataset

To train and test each ANN, we recorded sustained elbow flexion in 25 healthy young adults (mean ± SD, 22.6 ± 2.8 years). All subjects were right-handed, as determined via the Edinburgh Handedness Inventory [36]. The recorded dataset includes 7 static features: sex (9 female, 16 male), height (1.7 ± 0.07 m), mass (75.1 ± 12.2 kg), maximum elbow flexion torque (46.9 ± 14.0 Nm), forearm length (25.9 ± 1.7 cm), elbow width (9.5 ± 0.9 cm), and upper arm length (31.6 ± 2.5 cm). We also collected dynamic data, which included elbow flexion torques and EMG signals. Specifically, a Biodex System Pro 4 dynamometer (Biodex Medical, Shirley, NY) was used to record elbow flexion torques, comfortably constraining subjects to the same posture as targeted by our musculoskeletal simulations (Fig. 3b). We used ultrasound and the recommendations of [37] to guide the insertion of fine-wire electrodes to record EMG of the *brachialis*. Following SENIAM recommendations [38], we placed surface electrodes to record EMG of the *triceps*, *biceps*, and *brachioradialis*. Although more invasive, we used fine-wire EMG for the *brachialis* to circumvent the challenge of crosstalk with the *biceps brachii,* which could have presented during the high-intensity contraction. Elbow flexion torques were collected at 100 Hz, and EMG signals were collected at 3000 Hz. All EMG signals were checked for quality before, during, and after data collection.

During data collection, each subject performed two tasks using their non-dominant (i.e., left) arm: MVCs of elbow flexion and sustained elbow flexion. To obtain each subject’s elbow flexion strength, we recorded three 5-second MVC trials, each separated by 10 seconds of rest. Following the MVCs, each participant performed sustained elbow flexion with a target torque of 80% of their peak MVC torque. We selected this relatively high intensity to mitigate the influence of sex on time to task failure [33]. Similar to the protocol of Yoon et al. [33], the sustained elbow flexion trial was terminated when the subject’s torque dropped below 70% of their MVC torque for 3 of 5 seconds. During both tasks, subjects received verbal encouragement and real-time visual feedback of their torque plotted versus time.

Recorded EMG signals and elbow flexion torques were prepared for ANN training and testing. Each EMG signal was processed following the recommendations of [39]. First, we subtracted the mean of each signal from itself (i.e., centering about 0). Each signal was then processed through a 4^th^ order Butterworth bandpass filter with cutoff frequencies of 10–500 Hz and rectified. We then employed a 4^th^ order Butterworth lowpass filter (cutoff of 5 Hz) to retrieve the EMG envelope. We also employed a median filter (window size of 1499 instances, or 0.5 s) to remove any remaining outliers. The filtered signal was then scaled to a range of [0,1] using the peak muscle activation observed across all trials for the subject. Lastly, the processed muscle activations and elbow flexion torques were interpolated to the same length as the simulated trials (i.e., 300 time instances) across the length of the sustained elbow flexion trial. Sustained elbow flexion data from 5 randomly selected subjects formed the leave out set and the remaining 20 subjects’ data formed the training set.

### C. Direct Learning Model

We trained a custom ANN architecture to predict dynamic elbow flexion torques from recorded static (e.g., anthropometric measurements) and dynamic (i.e., muscle activations) features (Fig. 1, top). The recorded muscle activations were fed into a long short-term memory neural network (LSTM), a type of recurrent neural network capable of capturing long-term temporal dependencies, such as those embedded in muscle activations. We then concatenated this LSTM with a feedforward architecture, which learned to predict elbow flexion torques from the outputs of the LSTM and the 7 static subject-specific features. We regularized the ANN via weight decay, which penalized the weights and biases. A random search evaluated via mean-square-error (MSE) was used to identify the ANN’s hidden dimension and hyperparameters (i.e., learning weight and weight decay) in tandem with 5-fold cross validation. The ANN used a rectified linear unit (ReLU) activation function [40], an Adam optimizer [41], and a mean-square-error (MSE) loss function. The final architecture and hyperparameters of the direct learning model are summarized by Table II.

### D. Transfer Learning Model

To evaluate the utility of pre-training on simulated data, we developed an ANN via transfer learning (Fig. 1, bottom). The transfer learning model contained the same LSTM architecture as the direct learning model. However, this LSTM was pre-trained from scratch using the simulated dataset, predicting elbow flexion torque from muscle activations. By pre-training the LSTM with musculoskeletal simulations, we inform it of the relationship between muscle activations and elbow flexion torques, as captured by our simulations. During the pre-training phase, a random search informed the selection of learning rate and weight decay. Once trained, we locked the gradients of the LSTM’s hidden layers, and concatenated the model with a feedforward architecture. The resulting transfer learning model was then fine-tuned using the recorded dataset. During this fine-tuning, another random search was then used to identify the hyperparameters and feedforward architecture best suited to predict on recorded data. This random search also identified the number of LSTM backend layers to unfreeze, which was restricted to 0, 1, or 2 layers to prevent the model from re-training too many pre-trained layers. Similar to the protocol employed on the direct learning model, all random searches used in the development of the transfer learning model were evaluated via MSE in conjunction with 5-fold cross validation. Furthermore, the transfer learning model also employed a ReLU activation function [40], Adam optimizer [41], and MSE loss function. The final architecture and hyperparameters of the transfer learning model are summarized by Table II.

### E. Simulated Leave-Out Set

To provide a basis of comparison to a physics-based approach to predicting fatiguing elbow flexion torques, we ran a forward dynamic musculoskeletal simulation for each subject in the leave-out set. First, we scaled the generic elbow model to anthropometrically represent each of the 5 leave-out subjects. We then prepared the processed muscle activations for forward dynamic simulations. To reduce noise, we processed each signal using a moving average filter with a window size of 25 datapoints. This window size was determined by visualizing the sum of absolute differences for each signal (effectively quantifying alterations to the muscle activations), as processed through a moving average filter with window sizes ranging 5 to 50 datapoints, in 5 datapoint increments. Lastly, we input the processed muscle activations into forward dynamic simulations using the corresponding subject’s scaled musculoskeletal model, and evaluated the elbow flexion torques using a joint reaction analysis.

### F. Validation

To assess the impact of transfer learning with simulated and recorded data, we compared the performance of the direct learning model, transfer learning model, and musculoskeletal simulations on the leave out set. To summarize differences in the quality of predictions, we reported the root-mean-square-errors (RMSEs) and mean-absolute errors (MAE) for each ANN and simulation. Moreover, we visualized the raw predictions of each approach against the recorded data and created parity plots to elucidate the quality of predictions. Lastly, we compared the prediction variability from each ANN with the actual data’s standard deviation (SD), summarizing the smoothness of the predictions in comparison to their measured counterpart.

## III. RESULTS

**Fig. 4.**
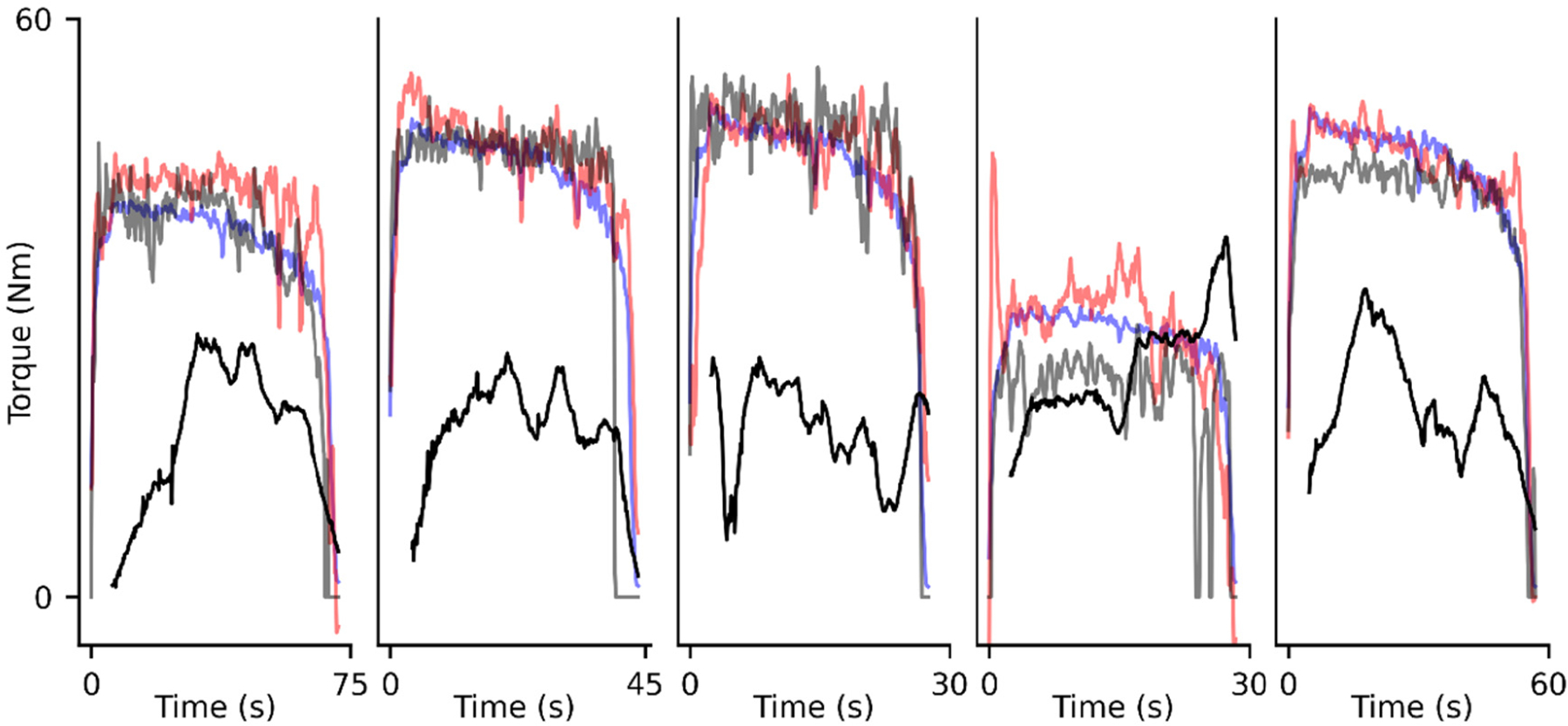
Recorded elbow flexion torques (grey), simulated elbow flexion torques (black), and predictions of the transfer learning model (blue) and direct learning model (red) over time. Each subplot corresponds to a subject in the leave-out set.

**Fig. 5.**
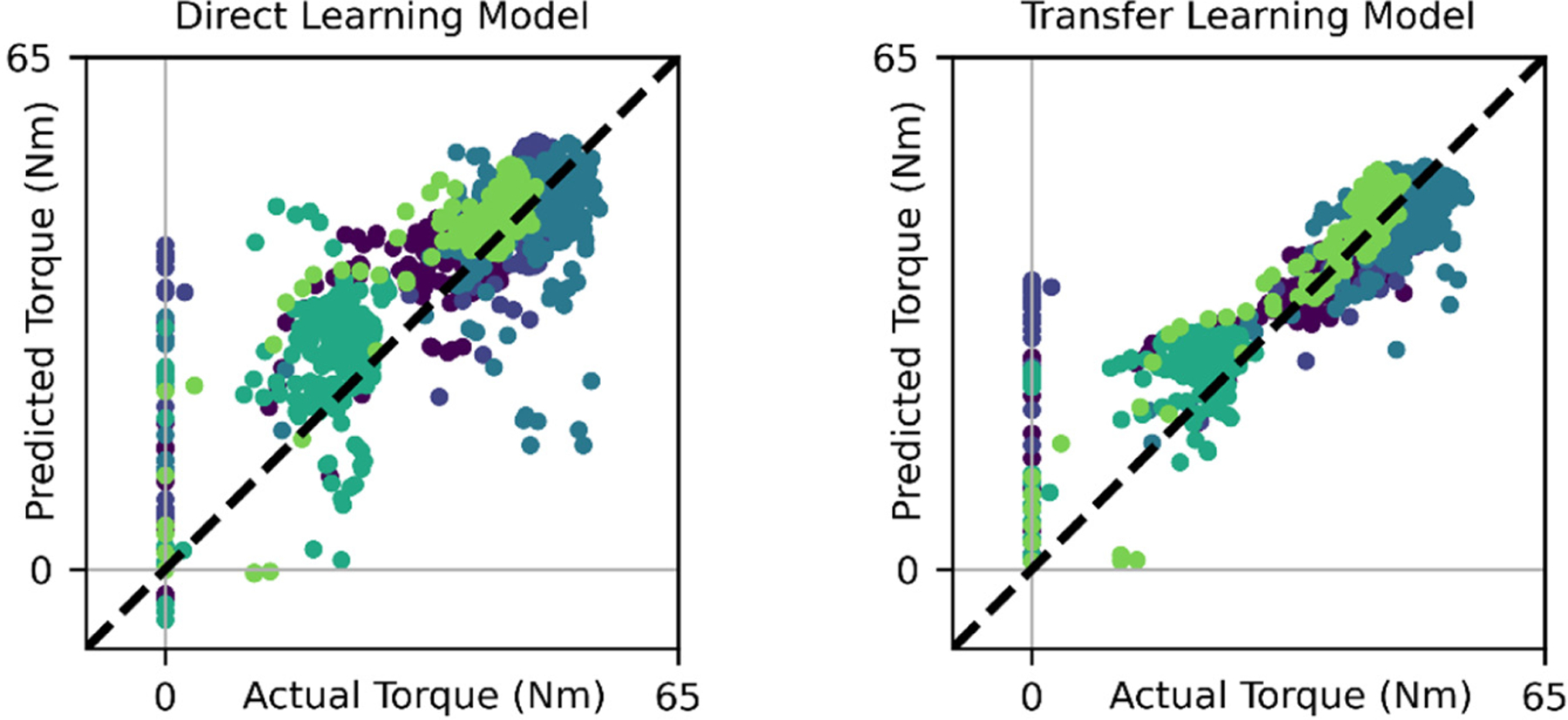
Parity plots of elbow flexion torques as predicted by the direct learning model (left) and transfer learning model (right) on leave-out data. Each color represents a subject. The transfer learning model consistently predicted with lower errors than the direct learning model, as illustrated by the comparably lower distances between each datapoint and the parity line for the transfer learning model. Both ANNs struggled to predict low torques (see aggregation of points where actual torque is 0 Nm).

The direct learning and transfer learning models predicted sustained elbow flexion torques from the leave out set with relatively little error (Fig. 4). The predictions from the transfer learning model and direct learning model produced RMSEs of 6.22 Nm and 8.28 Nm, respectively. This represents a 24.9% decrease in RMSE for the transfer learning model’s predictions when compared to the direct learning model. For context, as little as a 2.5 Nm change in elbow torque can indicate the effect of elbow laxity in baseball pitching mechanics [42]. Similarly, the MAEs associated with the transfer learning model and direct learning model were 4.09 Nm and 5.43 Nm, respectively. Both models substantially outperformed the musculoskeletal simulations, which consistently underpredicted elbow torques and produced RMSEs and MAEs of 57.9 Nm and 56.1 Nm, respectively. The errors for each approach can be interpreted in context of the recorded torque, which averaged approximately 37 Nm across the length of the task across all subjects. The transfer learning model’s predictions had comparable variability to those of the direct learning model, as reflected in their SDs of 10.0 Nm and 10.3 Nm, respectively (compared to a SD of 13.2 Nm for the recorded torque). Across all leave out subjects and both ANNs, the greatest prediction errors were associated with low elbow flexion torques, as is present during the both the ramp-up phase and the end of the task (Fig. 5).

**Fig. 6.**
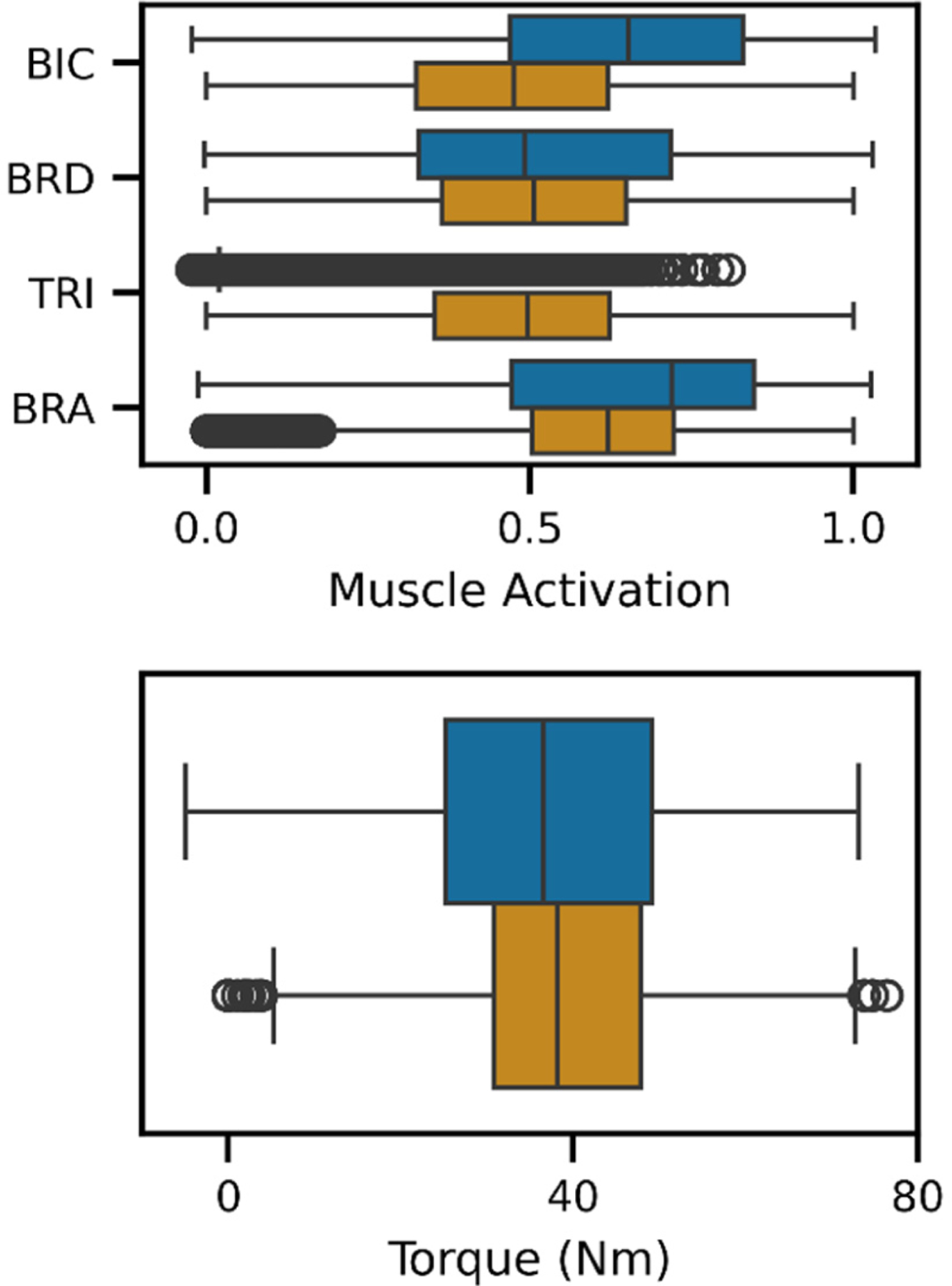
Boxplots of muscle activations and torques (ANN inputs and outputs, respectively) for the simulated (blue) and recorded (orange) datasets. Black circles indicate outliers, which were defined as values that fall below the first quartile or above the third quartile by 1.5 times the interquartile range. Distributions of the muscle activations and elbow flexion torques are similar across the two datasets, except for TRI muscle activations. Most of the simulated TRI muscle activations were at or near zero; only a subset of simulations converged to a solution with non-zero activation of the TRI, resulting in the outliers shown.

To supplement analyses of our transfer learning results, we characterized and compared the simulated (i.e., pretraining) and recorded (includes fine-tuning and test) datasets. Note, the simulated dataset was generated using synthetic subjects (representing healthy young adults) and forward dynamic simulations that received simulated muscle activations as inputs. The range of elbow flexion torques in the recorded and simulated datasets overlapped substantially with means and SDs of 38.2 Nm ± 13.9 Nm and 37.4 Nm ± 14.7 Nm, respectively. The distributions of muscle activations within each dataset were also alike (Fig. 6), especially for the elbow flexors (i.e., BIC, BRA, and BRD). However, the simulated TRI activations were much lower than those recorded. Additionally, the recorded torques increased in variability as each subject fatigued (i.e., near task failure), as shown at the end of each leave-out subjects’ trial (Fig. 4). For additional reference, the recorded elbow flexion MVCs were 53.0 Nm ± 14.8 Nm and the time to task failure (i.e., when a subject’s torque fell to 70% MVC) were 41.4 s ± 17.7 s, ranging 15.3 s to 89.8 s.

## IV. DISCUSSION

The direct learning and transfer learning models both predicted sustained elbow flexion torque with lower errors than the musculoskeletal simulations. Furthermore, the transfer learning model provided greater predictive performance than the direct learning model. In gist, pre-training the ANN with simulated data improved the model’s generalizability to recorded data. Even with the use of a relatively simple muscle fatigue model, our simulated dataset contains knowledge that is valuable for the prediction of sustained elbow flexion torque in healthy young adults. However, musculoskeletal simulations alone are not robust to real-world conditions, as evidenced by the poor predictive performance of these simulations on the leave-out set. Nonetheless, musculoskeletal simulations are a low-cost option for acquiring biomechanical data (in terms of time and money) and are widely available in open-source repositories (e.g., SimTK.org [43]). The direct learning and transfer learning models both struggled to predict at low elbow flexion torques; this outcome is likely the result of data imbalance, as more observations of high elbow-flexion torques were present in the training set. Future works may use simulated datasets and physics-based muscle fatigue models to pre-train other machine learning models, providing biomechanical predictions more robust to real-world conditions.

Transfer learning is most effective when the non-target dataset (i.e., data used to pre-train the model) is similar to that of the target dataset (i.e., data that the final model predicts). Prior works have noted this need for dataset similarity when transferring knowledge of non-target subjects to target subjects [44], [45]. Therefore, it is important that datasets used for training (non-target and target) be characterized when reporting transfer learning outcomes. Furthermore, augmenting the non-target dataset (e.g., using generative adversarial neural networks; [46]) may increase similarity with the target dataset, enhancing the benefits of transfer learning. Characterizations of our datasets revealed that the simulated and recorded data followed similar distributions. This similarity likely contributed to the success of our transfer learning approach, as indicated by the less erroneous predictions of the transfer learning model when compared to direct learning. Notably, the simulated dataset inherently lacked noise resultant from human subject data collection (e.g., movement artifact). The transfer learning model learned from these relatively clean data, potentially teaching it to predict smoother torque profiles.

The present work makes publicly available simulated and recorded datasets of elbow flexion. These data may be leveraged in future efforts to develop and validate models of fatiguing upper extremity movements. Our recorded dataset was collected using similar protocols to prior literature [33], [47], [48]. Similar to our recorded dataset, Hunter et al. [47] reported MVC torques of approximately 65 Nm ± 8 Nm for healthy young adults. Also consistent with this study, we observed an increase in torque variability near task failure (i.e., when subject was fatigued). However, the time to task failure previously reported for sustained elbow flexion at an 80% MVC torque target was previously reported at approximately 25 seconds [33], whereas we recorded a mean time to task failure of 41.4 seconds. This discrepancy could be the result of multiple factors, such as differences in the young adult population sampled, real-time feedback during the task, and shoulder posture (i.e., their subjects were positioned with 0° shoulder flexion, as opposed to 45° in the present study). Additionally, our simulated dataset provides muscle activations and torques representing 1,701 trials of elbow flexion across 243 healthy young adults which account for the onset of muscle fatigue. Using our datasets and nuanced machine learning techniques, such as multi-task learning [49] and explainable artificial intelligence [50], upper extremity models with even greater utility may be realized.

The simulation pipeline we employed is easily adaptable, making it a potentially fruitful tool for works seeking to model the effects of muscle fatigue. To pre-train our transfer learning model, we used a relatively simple 3-compartment muscle fatigue model. The simplicity of this fatigue model enabled its use with no subject-specific parameterization. However, future works may use other fatigue models to produce simulated datasets (e.g., that proposed by Callahan et al. [10]) with which to pre-train a transfer learning model. Using more robust fatigue models may increase the accuracy of musculoskeletal simulations, potentially providing even greater benefits for transfer learning.

The transfer learning approach we used is also adaptable, as training datasets and the ANN architecture can be adjusted as needed for developing deep biomechanical models. Our custom architecture enabled us to make predictions using our recorded multimodal data (e.g., time-series and static features). In doing so, our ANNs could capture the profiles of muscle activations while accounting for subject-specific attributes that are needed to predict elbow flexion torque. Similarly, many devices that could benefit from robust muscle fatigue models rely on muscle activations as inputs (e.g., myoelectric controllers). Future works may develop custom ANNs suited to predict the effects of fatigue using additional features (e.g., kinematic data), providing predictions for any of a wide range of biomechanical systems. Furthermore, the present study reports the benefits of transfer learning for predicting isometric elbow flexion torques. Future works may apply our framework to model other tasks, with consideration for any additional features needed to model that task (e.g., kinematic data for non-isometric tasks).

## V. CONCLUSION

By transfer learning from simulated to recorded datasets, we developed an elbow flexion model robust to the effects of muscle fatigue. Specifically, in this proof-of-concept study, we produced a model capable of capturing decreases in torque during a high-intensity sustained contractions in a healthy young adult population. Our results encourage the development of other transfer learning models, spanning beyond isometric elbow flexion. Through these efforts, models capable of capturing muscle fatigue may inform a wide variety of applications.

## Figures and Tables

**TABLE I:** Summary of Datasets Used to Train and Validate Direct Learning and Transfer Learning Models

		Simulated Dataset	Recorded Dataset
Trials		1701	25
Subjects		243	25
Features			
	Dynamic (muscle activations)		
		*Brachialis*	*Brachialis*
		*Triceps*	*Triceps*
		*Biceps*	*Biceps*
		*Brachioradialis*	*Brachioradialis*
	Static		
		N/A	Sex
			Height
			Mass
			Max Elbow Flexion Torque
			Forearm Length
			Elbow Width
			Upper Arm Length
Outputs			
	Dynamic		
		Elbow Flexion Torques	Elbow Flexion Torques

**TABLE II:** Architecture and Hyperparameters of the Direct Learning and Transfer Learning Models

	Direct Learning	Transfer Learning
Learning Rate	6.4 × 10^−3^	9.0 × 10^−3^
Weight Decay	4.5 × 10^−2^	9.4 × 10–2
LSTM Hidden Layers	3	3
LSTM Hidden Layer Width	24	24
Backend LSTM Layers Unfrozen	N/A	2
Feedforward Hidden Layers	2	2
Feedforward Hidden Layer Width	_23_	18
